# S02-3 Physical activity policies and good practices in Europe

**DOI:** 10.1093/eurpub/ckac093.008

**Published:** 2022-08-29

**Authors:** Romeu Mendes, Stephen Whiting, Ana Barbosa, Keeva Duffey, Adriana Pinedo, Patricia Arnaiz, Kremlin Wickramasinghe

**Affiliations:** 1 Division of Country Health Programmes, WHO Regional Office for Europe, Copenhagen, Denmark; 2 EPIUnit – Instituto de Saúde Pública, Universidade do Porto, Porto, Portugal

**Keywords:** Physical activity, policies, Europe, interventions

## Abstract

**Background:**

The establishment of the EU physical activity (PA) guidelines and the HEPA monitoring framework has had an impact on policy development and implementation across the region from 2015. This works presents results from the third round of monitoring in 2021 and discusses trends since 2015.

**Methods:**

A questionnaire was distributed in 2021 to all EU Member States of the WHO European Region through the network of PA Focal Points, who were requested to collect data from national colleagues and complete the questionnaire. All EU Member States (27 in 2021) responded to the survey on the implementation of the 23 indicators of the HEPA monitoring framework.

**Results:**

The results of the 2021 round of data collection on HEPA indicators showed an overall stabilization of the implementation of PA promotion policies. Besides important increases in several indicators, such as indicators 15 (HEPA in the training of physical education teachers), 20 (Schemes to promote physical activity at the workplace) and 21 (Schemes for community interventions to promote physical activity in older adults), many others decreased and others showed no progress. Most national physical activity policies or action plans were multi-sectoral, with good coverage of the sectors recognized as important for HEPA promotion. While some methodological aspects may have affected the results, this round also reflected policy implementation during the COVID-19 pandemic (2019–2021). COVID-19 has had a significant impact on all sectors of society but especially on health, sports, education and mobility, which are major areas for PA promotion and policy implementation.

**Conclusions:**

There seems to be an overall stabilization of the implementation of PA promotion policies since 2015. Public health experts and decision makers could utilise the increase in public awareness of the health benefits of physical activity kindled by the COVID-19 crisis to implement new health-promoting policies. Policy design, development and implementation of HEPA promotion must be strengthened for post-COVID-19 social and economic recovery.

